# The Association between Air Pollution and Subclinical Atherosclerosis: Rivera et al. Respond

**DOI:** 10.1289/ehp.1307403R

**Published:** 2014-01-01

**Authors:** Marcela Rivera, Xavier Basagaña, Inmaculada Aguilera, Maria Foraster, David Agis, Eric de Groot, Laura Perez, Michelle A. Mendez, Laura Bouso, Jaume Targa, Rafael Ramos, Joan Sala, Jaume Marrugat, Roberto Elosua, Nino Künzli

**Affiliations:** 1 University of Montreal Hospital Research Centre (CRCHUM), Montreal, Quebec, Canada; 2 Centre for Research in Environmental Epidemiology (CREAL), Barcelona, Spain; 3 Hospital del Mar Research Institute (IMIM), Barcelona, Spain; 4 CIBER Epidemiología y Salud Pública (CIBERESP), Spain; 5 Universitat Pompeu Fabra, Barcelona, Spain; 6 Department of Vascular Medicine, Academic Medical Centre, Amsterdam, the Netherlands; 7 Swiss Tropical and Public Health Institute, Basel, Switzerland; 8 University of Basel, Basel, Switzerland; 9 University of North Carolina at Chapel Hill, Chapel Hill, North Carolina, USA; 10 4sfera Innova, Girona, Spain; 11 Research Unit, Family Medicine, Jordi Gol Institute for Primary Care Research (IDIAP Jordi Gol), Catalan Institute of Health, Catalunya, Spain; 12 Department of Medical Sciences, School of Medicine, University of Girona, Girona, Spain; 13 Servicio de Cardiología, Hospital Universitari Josep Trueta, Institut Català de la Salut, Girona, Spain; 14 Grupo de Epidemiología y Genética Cardiovascular, Hospital del Mar Research Institute (IMIM), Barcelona, Spain

We appreciate Kawada’s interest in our study (Rivera et al. 2013). His letter focuses on some limitations of our study, which were already discussed in our article.

Kawada notes that the small number of participants with low ankle–brachial index (ABI) provided low power to detect an association; this is a limitation that we acknowledged in our article (Rivera et al. 2013). Kawada also points out that the number of covariates in the model exceeds the common rule of thumb of having at least 10 events per variable. We recognize that, with a small number of cases, one is inevitably faced with the trade-off between including all relevant confounders and keeping the number of covariates to a minimum. However, in our article we provided results from a minimally adjusted model including only five confounders and an interaction term. In this model, the number of events per variable was > 10, and the results were not significantly different from those of the fully adjusted model. Kawada suggests selecting a “higher cut-off value of ABI, such as 1.0” for low ABI given that “an ABI of 0.9–1.0 is also associated with cardiovascular risk (Ono et al. 2003).” The findings of Ono et al. were for patients on hemodialysis due to end-stage renal disease and thus cannot be extrapolated to healthy population samples such as the one considered in our study (i.e., with no history or current signs of cardiovascular disease). We selected a cut-off value of 0.9 for low ABI because of the strong evidence of increased risks of incident cardiovascular disease, morbidity, and mortality in individuals with ABI < 0.9 (Allison et al. 2008; Ankle Brachial Index Collaboration 2008; Lee et al. 2004; McDermott et al. 2005). A cut-off value of 0.9 is also more specific (Lee et al. 2004) and much more common in the literature, which allows comparison with other studies.

Kawada’s second argument involves the consideration of multicollinearity in the fully adjusted model (model 2), in which systolic and diastolic blood pressure were included. Systolic and diastolic blood pressure were only moderately correlated (correlation coefficient, 0.62), and according to the variance inflation factor (VIF), there were no multicollinearity problems (VIF was 2.45 for systolic blood pressure and 1.98 for diastolic blood pressure).

Kawada points at “contradictory results” in the association between air pollution and carotid intima media thickness (IMT), mainly based on the null and weak associations found by Lenters et al. (2010) between several markers of air pollution [nitrogen dioxide, sulfur dioxide, PM_2.5_ (particulate matter ≤ 2.5 μm in aerodynamic diameter), black smoke, and traffic intensity] and three indicators of vascular damage (IMT, pulse wave velocity, and augmentation index). We consider, however, that these do not constitute results contradictory to the positive association between air pollution and subclinical markers of atherosclerosis found in the six studies thoroughly discussed in our article (Bauer et al. 2010; Diez Roux et al. 2008; Hoffmann et al. 2007, 2009; Künzli et al. 2005, 2010) as well as by Wilker et al. (2013). The study by Lenters et al. (2010) involved a cohort of young adults, on average 28 years of age. Exposure to air pollution was estimated at the current address only. As the authors acknowledged, the young age of participants and the exposure misclassification, which resulted from exposure estimated at the current address only, are likely explanations for their mixed results.

Finally, we agree with Kawada’s closing remark on the need for longitudinal studies, as we concluded in our article.
